# Supplementary material for: Bi-enzymes treatments attenuate cognitive impairment associated with oxidative damage of heavy metals
Source: R Soc Open Sci. 2021 Jan 13;8(1):201404. doi: 10.1098/rsos.201404 (PMC7890482; doi:10.1098/rsos.201404)
Supplement: Figures S1 - S3 [file rsos201404supp1.docx]

Supporting information

Bi-Enzymes Treatments Attenuate Cognitive Impairment Associated with Oxidative Damage of Heavy Metals

Chao Chen^1†^, Xiaoxin Zhang^2†^, Hao Huang^1^, Hongyi Bao^1^, Xiaodong Li ^1^, Ye Cheng^4^, Jing Zhang^1^, Yin Ding^5^, Yanguang Yang^3*^,Haiying Gu^1*^ ,Donglin Xia^1*^

^1^ School of Public Health, Nantong University, Nantong, Jiangsu, 226019, China.

^2^ Boao Evergrande International Hospital, Qionghai, Hainan, 571400, China.

^3^ Nantong Tumor Hospital, Nantong, Jiangsu, 226361, China.

^4^ Xinglin college, Nantong university, Nantong, Jiangsu, 226019, China

^5^ State Key Laboratory of Analytical Chemistry for Life Science, School of Chemistry and Chemical Engineering, Nanjing University, Nanjing, Jiangsu 210093, P. R. China

**Keywords:** Cognitive impairments, Oxidative stress, Morris Water Maze, Lead, Bi-enzymes

1.Method

**Hemolysis assay.** Hemolytic activity of PLGA@SOD-CAT and its components was tested against rat red blood cells (RBCs). Briefly, Fresh blood collected from SD rats was centrifuged and washed three times with PBS. RBCs suspended in PBS or distilled water were set as negative control (0% hemolysis), or positive control (100% hemolysis). A suspension of RBCs (4%, v/v) was mixed with SOD (19.87 μg/mL), CAT (24.67 μg/mL), or PLGA@SOD-CAT (0.1 mL) for 1 h at 37°C and then centrifuged. The optical density of the supernatants at 450 nm (OD 450) was measured by a microplate reader (BioTek microplate reader). The hemolytic activity was quantified according to the following formula:

Hemolysis (%) = [(OD sample– OD negative control)/(OD positive control – OD negative control)] × 100%.

**Cytotoxicity assay.** Cytotoxic activity of the peptides was assessed against endothelial cell line. Cells (1 × 105 per well) were seeded into 96-well microplates and incubated with different amount of PLGA@SOD-CAT for 24 h. Cell viability was monitored with CCK8 kit (CCK8, Beyotime Institute of Biotechnology, Haimen, China) following the producers suggestions.

**Morris water maze.** The 25 rats were randomly divided into Five groups: (1) Control, administrated with 0.1 mL PBS via the *tail vein* at the first day. (2) Pb^2+^, administrated with 6.5 mg/kg/d lead nitrate for five days. (3) Free SOD/CAT group, administrated with 6.5 mg/kg/d lead nitrate for five days, and 1.99 μg SOD, 2.47 μg CAT via the *tail vein* at the first day. (4) PLGA without SOD or CAT, administrated with 6.5 mg/kg/d lead nitrate for five days and 0.1 mL PLGA via the *tail vein* at the first day. (5) PLGA@SOD-CAT group, administrated with 6.5 mg/kg/d lead nitrate for five days and 0.1 mL PLGA@SOD-CAT (with SOD 19.87 μg/mL, CAT 24.67 μg/mL) via the *tail vein* at the first day.

The water maze consisted of an off-white circular pool (80 cm in diameter) with the upper part surrounded by a 40-cm-high Perspex wall and filled with water at 25 ± 1 °C [29]. The circular pool was segmented into four quadrants and enclosed in a curtain. The motions caused by rats were recorded by a camera. The training procedure included four trials each day with four different starting positions from 16th to 20th day. In each trial, rats were allowed to find the escape platform from the center of the south-west quadrant of the pool within 90 seconds. All rats were allowed to rest on the platform for 15-20 seconds after the platform was mounted. Thereafter, the rats were led to the platform and kept for 15 seconds. The hidden platform task (spatial reference memory) was performed on the 21th day. The escape latency, escape length, swimming speed, and rounds at which the rats crossed the platform was recorded.

2. Results


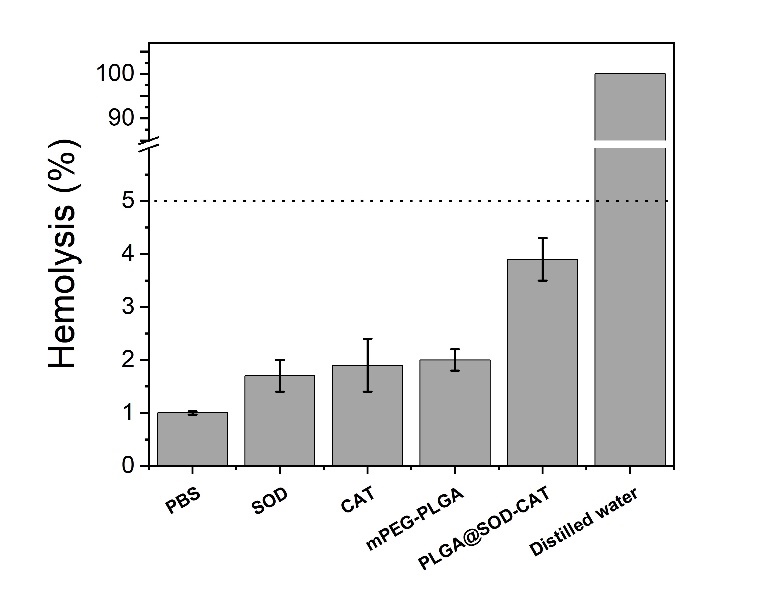


**Figure S1.** Hemolytic activity of SOD (19.87 μg/mL), CAT (24.67 μg/mL), and PLGA@SOD-CAT (0.1 mL) on rat erythrocytes after 1 h of incubation at 37°C (n=5). The hemolytic activity was evaluated by the spectrophotometric determination of hemoglobin released from erythrocytes. PBS (0% hemolysis) and distilled water (100% hemolysis) were used as controls. Hemolysis values ≤ 5% (dashed line) is considered to be non-hemolytic.


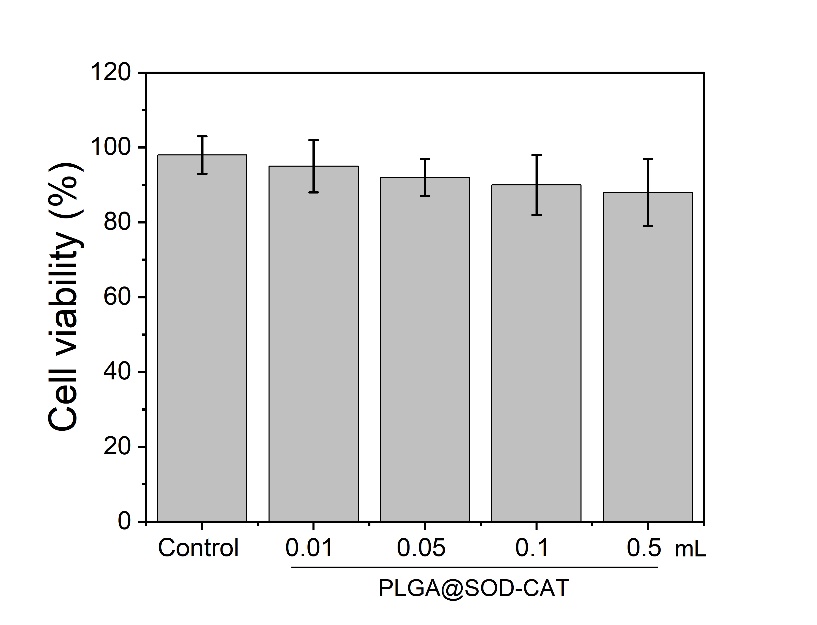


**Figure S2.** Cytotoxicity of PLGA@SOD-CAT against the endothelial cell line after incubating for 24 h (n = 5). PLGA@SOD-CAT did not exhibit a significant cytotoxic effect toward endothelial cells at any of the tested concentrations.


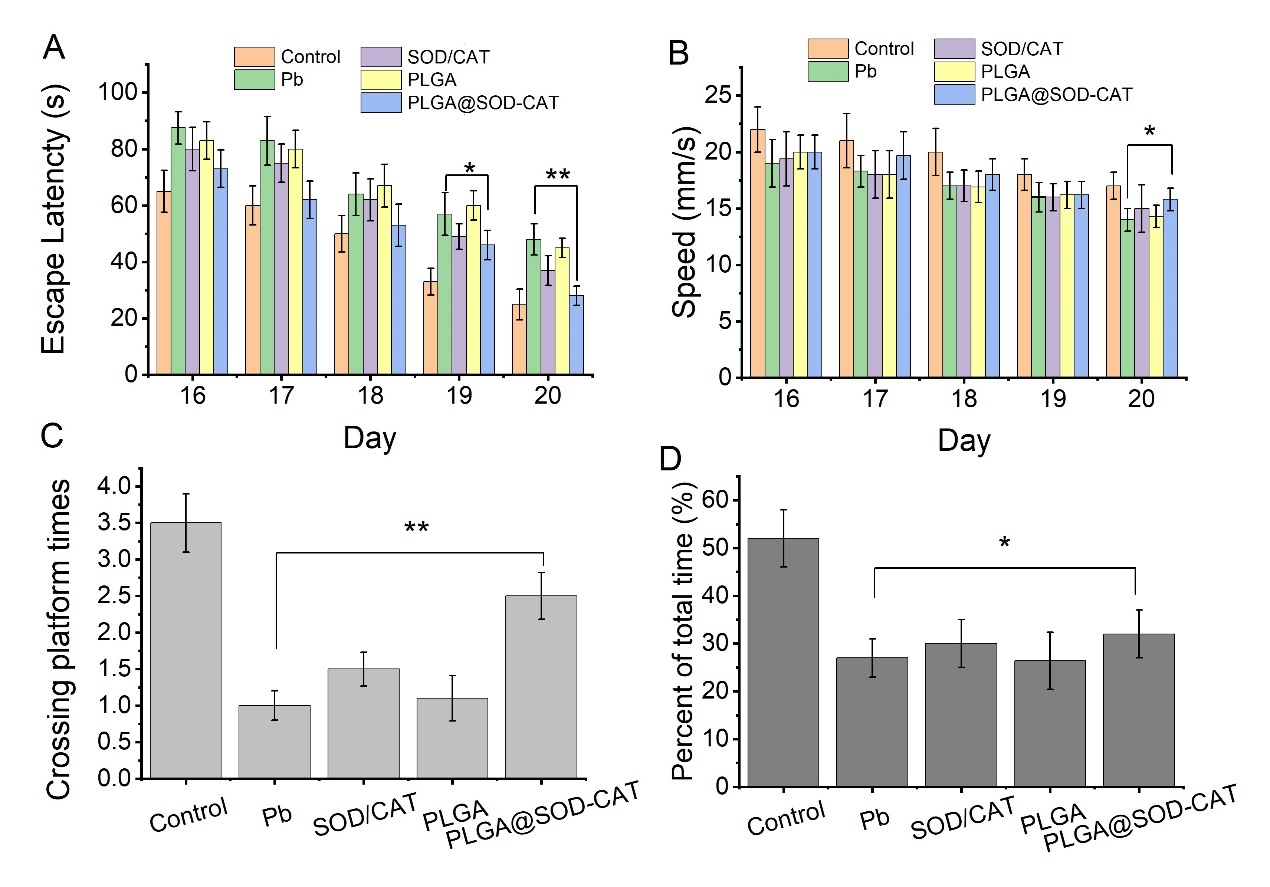


**Figure S3.** Effects of different groups on spatial learning task impairment and memory loss in young rats using the Morris water maze test. (A) The escape latencies of rats in the spatial learning task during the four training days. (B) Motion speed of rats in the probe test. (C) Crossing platform times of rats in the test. (D) Time percent during the platform quadrant in the probe test. * *P* < 0.05, ** *P* < 0.01.
